# Health-Related Quality of Life in Adolescents and Young Adults with Congenital Heart Disease and Its Predictors, Including Subjective Transition Readiness to Adulthood

**DOI:** 10.1007/s00246-025-04033-x

**Published:** 2025-09-20

**Authors:** Kazuteru Niinomi, Shoji Yoshikawa, Toshiaki Sato, Hiroko Omori, Kayoko Miyashita

**Affiliations:** 1 Department of Integrated Health Sciences, Nagoya University Graduate School of Medicine, 1-1-20 Daiko-Minami, Higashi-ku, Nagoya, Aichi 461-8673 Japan; 2 Faculty of Nursing, Shijonawate Gakuen University, Daito, Osaka Japan; 3 College of Nursing, Gifu College of Nursing, Hashima, Gifu Japan; 4 Graduate School of Nursing, Osaka Metropolitan University, Osaka, Japan

**Keywords:** Adolescents and young adults, Congenital heart disease, Health-related quality of life, Self-reliant life, Transition

## Abstract

Although international studies have examined the health-related quality of life (HRQoL) of adolescents and young adults (AYAs) with congenital heart disease (CHD), those focusing specifically on predictors including transition readiness to adulthood remain limited, particularly in Japan. This study aimed to evaluate HRQoL among AYAs with CHD and its predictors. This cross-sectional survey included 86 participants with CHD from 4 outpatient pediatric cardiology clinics and a parents’ peer support group in Japan. The MOS Short-Form 36-Item Health Survey (SF-36) was conducted, and information was obtained on demographics, physical conditions, and participants’ transition readiness assessment. The three SF-36 component summary scores as dependent variables were compared against standard values and analyzed using multiple linear regression analyses. Participants’ mean age was 21.3 years (standard deviation = 3.6). Compared to national standard values, component scores showed no apparent differences, except in a lower role/social component summary in females. The presence of arrhythmia and cyanosis, New York Heart Association (NYHA) functional classification, physical disability certification grade, and a transition readiness item together accounted for 36.1% of the variance in the physical component summary. Families living together, CHD-related surgery experience within the last 3 years, and one transition readiness item explained 19.8% of the variance in the mental component summary. Participants’ sex and NYHA explained 13.6% of the variance in RCS. Physical conditions and subjective transition readiness partially predicted the HRQoL of AYAs with CHD. Females may be aware that being unable to fulfill expected social roles may prevent them from achieving optimum HRQoL.

## Introduction

Congenital heart disease (CHD) is the most common congenital disorder, with a global prevalence of 8.2 per 1000 live births [1]; it accounts for approximately one-third of all major congenital anomalies [2]. Although ventricular septal defect and atrial septal defect are the most prevalent lesions, patients requiring long-term specialized care are more likely to present with moderate-to-complex lesions such as tetralogy of Fallot, transposition of the great arteries, and single-ventricle physiology after Fontan circulation [3]. Children with CHD experience significantly higher mortality, particularly within the first 4 years of life [3]; however, advances in surgery and medical care have markedly improved survival. In high-income countries, over 90% of children with CHD now survive into adulthood [4, 5]. In the United States, approximately 80% reach 35 years of age [6]. In Japan, 9000–10,000 CHD surgeries are performed annually with a reported survival rate of 95%, resulting in an estimated 9000 new adult survivors each year [7, 8]. However, CHD continues to be a chronic and potentially fatal condition that requires continuous, individualized care. Patient- and family-centered approaches are increasingly recognized as essential for improving outcomes [9–12].

As survival improves, adolescents and young adults (AYAs) with CHD face unique challenges when transitioning from pediatric to adult care [13–15]. Structured transition programs have been shown to improve disease-specific knowledge, enhance self-management skills, and reduce loss to follow-up [16–18]. Tools such as the Transition Readiness Assessment Questionnaire (TRAQ) [19], a validated patient-reported outcome measure (PROM), have been used in populations with chronic illnesses, including cardiovascular diseases [19]. Uzark et al. pioneered the assessment of transition readiness in AYAs with CHD using study-specific instruments based on existing generic tools [12, 20]. However, there is no validated, standardized CHD-specific transition readiness instrument, particularly a Japanese version [21, 22]. According to international guidelines, transition should promote medical independence as well as social participation and self-reliance [23]. However, relatively few studies have examined the health-related quality of life (HRQoL) of AYAs with CHD in relation to their readiness for transition. A meta-analysis of 18 observational studies on AYAs with CHD involving 1786 participants found no overall difference in their HRQoL compared with controls; however, physical HRQoL was lower [24]. Similarly, a Portuguese survey reported that AYAs with CHD experienced better HRQoL in the environmental and social relationship domains compared with controls, whereas those who underwent surgery had lower physical and social HRQoL [25]. In Japan, studies on adults with CHD revealed lower HRQoL than that for the general population, with consistently lower physical component summary (PCS) scores than mental component summary (MCS) scores [26]. However, these studies did not report on the role/social component summary (RCS) in the Japanese version of the MOS 36-Item Short-Form Health Survey (SF-36) [27]. Moreover, evidence of sex differences in vocational opportunities and employment suggests the importance of considering sex when developing individualized transition plans [28].

PROMs, such as the SF-36, are increasingly used to inform patient-centered care and health policy [29, 30]. Although the Japanese version of SF-36 includes a culturally adapted RCS domain, this aspect has not been adequately examined in previous studies of AYAs with CHD. Despite the growing importance of PROMs, consensus on HRQoL in this population remains limited, and predictors of HRQoL, including subjective transition readiness, are poorly defined. Therefore, the present study aimed to evaluate HRQoL among Japanese AYAs with CHD using the SF-36 and identify its predictors, with particular focus on the subjective perceptions of transition readiness.

## Materials and Methods

### Participants and Procedure

This cross-sectional study included 86 AYAs with CHD, recruited from four pediatric cardiology outpatient clinics and a parent peer support group in western Japan. Eligibility criteria were as follows: (1) aged 16–29 years, (2) diagnosed with CHD, (3) graduated from junior high school, and (4) regularly attending outpatient follow-up. Individuals judged unable to complete a self-administered questionnaire due to intellectual or physical disability were excluded. Data were collected between February 2017 and December 2018. This study was conducted in accordance with the Declaration of Helsinki and approved by the institutional review boards of Osaka University Hospital (No. 16412, approved February 13, 2017) and Shijonawate Gakuen University (No. 2017005, approved January 1, 2018). Written informed consent was obtained from all participants. For minors, parental notification and an opt-out procedure were used. In Japan, pediatric cardiology services often extend into the early to late 20s, depending on individual needs and institutional practices [16, 18]. Although transition to adult care is generally recommended around age 18–20 years, the process is frequently gradual.

## Measures

### Participant Characteristics

Demographic and clinical data included the following: age, sex, cohabitation with family, surgical and hospitalization history related to CHD, presence of subjective symptoms (arrhythmia and cyanosis), transition status to adult care, New York Heart Association (NYHA) functional classification [31], and certification of physical disability [32]. Based on an inter-investigator discussion and a systematic review of children who had undergone open-heart surgery over at least 2 years and were followed up [33], information on any CHD-related hospitalizations or surgery in the past 3 years was obtained. The NYHA functional class, a subjective estimate of a patient’s functional ability [31], has the following classifications: Class I, no limitation; Class II, slight limitation; Class III, marked limitation; and Class IV, unable to perform any physical activity. Certification of physical disability utilizes the following certified grades: Grade 1 = extremely restricted activities of daily living, Grade 2 = significant restriction on daily life at home, and Grade 3 = significant restriction on daily life in society, based on the Act for the Welfare of Persons with Disabilities in Japan [32].

### HRQoL

HRQoL was assessed using the Japanese version of SF-36 version 2 (SF-36v2) [27, 34, 35]. The SF-36, used internationally as a self-administered questionnaire, consists of 36 items in the following eight domains: physical functioning, role physical, bodily pain, general health perception, vitality, social functioning, role emotional, and mental health [22]. These eight domains were recategorized into a three-component model in the Japanese version, consisting of PCS, MCS, and RCS [27, 36]. The scores for each domain and component were calculated on a scale of 0–100 according to population norm-based scoring using the Japanese standard value of 50 (SD = 10) [27]. Although higher scores indicate better HRQoL, scores < 50 indicate a poorer component or domain than the national norm.

### Transition Readiness to a Self-Reliant Adulthood Life

To assess participants’ subjective transition readiness to self-reliant adulthood, we developed two overarching items based on a literature review, existing checklists such as the TRAQ, and consensus among pediatric nursing researchers: (1) “I take care of myself independently while receiving support” and (2) “I am living my own life despite my illness.” Each item, scored on a 4-point Likert scale, had the following responses: 1 “Not at all,” 2 “Slightly,” 3 “Very,” and 4 “Definitely.” These items were conceptualized to capture the core conceptual elements of transition readiness, self-management, and autonomous living, while minimizing respondent burden. Although transition readiness has been assessed using several generic instruments (e.g., TRAQ) in populations with chronic illnesses, there is currently no validated, standardized measure developed specifically for AYAs with CHD, and no Japanese version has been established [12]. Therefore, in the present study, we included brief, subjective items to capture transition-related perceptions relevant to this population.

## Data Analysis

Descriptive statistics were used to summarize background characteristics. Additionally, the SF-36 eight domains and three components’ scores were calculated and compared with Japanese national standard values [27]. Univariate analysis was performed to determine the correlations among background characteristics, participants’ self-reported readiness to transition to adulthood, and three SF-36 components’ scores. For the univariate analysis, all nominal and ordinal variables were binarized: sex (male = 1, female = 0); presence of family living together (yes = 1, no = 0); experienced CHD surgery within the last 3 years (yes = 1, no = 0); experienced hospitalization within the last 3 years (yes = 1, no = 0); arrhythmia (already started = 1, no = 0); cyanosis (yes = 1, no = 0); starting transition from pediatrics to adult outpatient (yes = 1, no = 0); NYHA classification (had no limitation in physical activity = 1, had limitation = 0); certification of physical disability (certified = 1, not certified = 0); age (teen = 1, over 20 = 0); and subjective transition readiness to adulthood from the two questionnaires’ results (definitely or very = 1, slightly or not at all = 0). Each variable that was significantly correlated with one of the three SF-36 components’ scores was considered an independent variable and included in multiple linear regression analysis (using the stepwise method), with the three SF-36 components’ scores as dependent variables. Multicollinearity was examined using tolerance values and variance inflation factors. All statistical analyses were performed using IBM SPSS Statistics for Windows, version 21 (IBM Corp., Armonk, NY, USA). Post hoc analysis indicated adequate-to-moderate power (0.84–0.67) for each regression model, assuming an effect size of 0.15 and a significance level of 0.05 [37, 38]. Using a two-tailed test, the level of significance was set at *p* < 0.05.

## Results

### Demographic and Background Characteristics (Table 1)

**Table 1:** Participant background characteristics

Variables	*M* (*SD*)	*n* (%)
Sex		
Male		43 (50.0)
Female		43 (50.0)
Age (years)	21.3 (3.6)	
16–19		31 (36.0)
20–30		55 (64.0)
Cohabitation with family		
Yes (multiple answers)		71 (82.6)
Living with a partner		3 (3.4)
Living with a father		53 (61.6)
Living with a mother		65 (75.6)
Living with children		2 (2.3)
Others (living with)		34 (39.5)
No		15 (17.4)
Experienced CHD surgery		
Yes		82 (95.3)
Finally within the last 3 years		13 (15.9)
Not finally within the last 3 years		62 (75.6)
N/A		7 (8.5)
No		4 (4.7)
Experienced hospitalization related to CHD		
Yes		82 (95.3)
Finally within the last 3 years		13 (15.9)
Not finally within the last 3 years		62 (75.6)
N/A		7 (8.5)
No		4 (4.7)
Subjective symptoms		
Arrhythmia		
Yes		29 (33.7)
No		52 (60.5)
N/A		5 (5.8)
Cyanosis		
Yes		9 (10.5)
No		72 (83.7)
N/A		5 (5.8)
Transition from pediatrics to adult outpatient		
Yes, I have already started		17 (19.8)
No, I have not started yet		66 (76.7)
N/A		3 (3.5)
Functional classification of NYHA		
I		51 (59.3)
II		26 (30.2)
III		1 (1.2)
IV		2 (2.3)
N/A		6 (7.0)
Certification of physical disability		
Grade 1		25 (29.1)
Grade 3		16 (18.6)
Grade 4		7 (8.1)
Not certified		28 (32.6)
N/A		10 (11.6)

*N* = 86

*CHD* congenital heart disease, *N/A* not applicable, *NYHA* New York Heart Association, *M* mean, *SD* standard deviation

The 86 included participants’ mean age was 21.3 years (SD = 3.6, range: 16–30), with half each of males and females (Table 1). Over 95% had undergone CHD-related surgery and hospitalization. Regarding the transition status, only 17 (19.8%) participants had initiated a transition to adult outpatient care, whereas most (76.8%) had not yet begun. Therefore, some participants were receiving both pediatric and adult cardiology care services simultaneously.

### SF-36 Scores (Table 2)

**Table 2:** SF-36 scores

SF-36 domain/component scores	*M* (*SD*)			
	Male	Standard value for males	Female	Standard value for females
	(*n* = 43)	(*n* = 1113)	(*n* = 43)	(*n* = 1166)
Physical functioning	50.0 (10.0)	51.0 (9.5)	48.1 (9.6)	49.0 (10.5)
Role physical	50.1 (7.7)	50.5 (9.9)	44.0 (12.4)	49.5 (10.1)
Body pain	57.3 (7.2)	51.0 (9.8)	50.8 (11.3)	49.0 (10.2)
General health	48.7 (9.3)	50.1 (10.0)	48.3 (10.5)	49.9 (10.0)
Vitality	50.8 (8.6)	50.5 (9.8)	45.4 (12.7)	49.5 (10.2)
Social functioning	52.7 (7.3)	50.8 (9.6)	45.6 (12.6)	49.2 (10.4)
Role emotional	50.2 (7.2)	50.6 (9.7)	45.5 (10.7)	49.4 (10.3)
Mental health	52.0 (10.0)	50.3 (9.7)	46.6 (11.9)	49.7 (10.3)
Physical component summary (PCS)	50.7 (10.0)	50.7 (9.4)	50.5 (9.4)	49.3 (10.2)
Mental component summary (MCS)	52.0 (7.1)	50.2 (9.7)	48.3 (12.0)	49.8 (9.9)
Role/social component summary (RCS)	50.5 (9.6)	50.6 (10.3)	43.7 (10.9)	49.4 (10.7)

Scores indicate Norm-Based Scoring (mean = 50, standard deviation = 10)

*M* mean, *SD* standard deviation, *SF-36* the short form 36-item health survey

Compared to the national standard values by sex, no apparent differences were observed in the three-component summary scores, except for RCS, which was notably lower among females (Table 2). In the eight SF-36 domains, bodily pain scores exceeded the standard value in both sexes, though the increase was more pronounced in males. Among males, general health scores were approximately equivalent to the standard value (≤ 1 point difference). In contrast, females scored lower than the standard value in seven domains: vitality, social functioning, role emotional, and mental health, with a difference of ≥ 3 points. Social functioning scores were higher than the standard values in males but lower in females. While no notable sex difference was seen in PCS scores, females scored ≥ 5 points lower in RCS, and MCS scores were higher in males than in females.

### Subjective Transition Readiness to Adulthood (Table 3)

**Table 3:** Subjective transition readiness to adulthood

Variables	*n* (%)
I take care of myself independently while receiving support	
Definitely	35 (40.7)
Very	35 (40.7)
Slightly	10 (11.6)
Not at all	1 (1.2)
N/A	5 (5.8)
I am living my own life despite my illness	
Definitely	53 (61.6)
Very	26 (30.2)
Slightly	6 (7.0)
Not at all	0 (0)
N/A	1 (1.2)

*N* = 86

*N/A* not applicable

Most participants responded “definitely” or “very” to both transition readiness items: “I take care of myself independently while receiving support” and “I am living my own life despite my illness” (Table 3).

### Univariate Analysis (Table 4)

**Table 4:** Univariate linear regression analysis on the scores of three SF-36 components

Independent variables	*B*	*β*	95% CI		*p*
			Lower	Upper	
Dependent variable: physical component summary (PCS)					
Sex	− 0.21	− 0.01	− 4.40	3.97	0.919
Presence of family living together	− 6.00	− 0.24	− 11.35	− 0.64	0.029
Experienced surgery of CHD within last 3 years	− 2.02	− 0.08	− 8.04	4.01	0.507
Experienced hospitalization within the last 3 years	− 4.97	− 0.25	− 9.37	− 0.57	0.027
Arrythmia	− 4.97	− 0.25	− 9.37	− 0.57	0.027
Cyanosis	− 7.19	− 0.35	− 11.52	− 2.87	0.001
Starting transition pediatrics to adult	1.22	0.05	− 4.12	6.59	0.852
NYHA	6.17	0.30	1.76	10.58	0.007
Certification of physical disability	− 9.36	− 0.49	− 13.05	− 5.67	< 0.001
Age	2.41	0.12	− 1.91	6.74	0.027
I take care of myself independently while receiving support	9.48	0.33	3.42	15.54	0.003
I am living my own life despite my illness	3.61	0.96	− 4.57	11.78	0.383
Dependent variable: mental component summary (MCS)					
Sex	− 3.67	− 0.18	− 7.91	0.58	0.090
Presence of family living together	5.64	0.22	0.08	11.19	0.047
Experienced surgery of CHD within last 3 years	− 8.20	− 0.30	− 14.34	− 2.07	0.009
Experienced hospitalization within the last 3 years	− 2.69	− 0.13	− 7.50	2.13	0.270
Arrythmia	0.67	0.03	− 3.72	5.06	0.762
Cyanosis	− 5.92	− 0.20	− 12.49	0.64	0.076
Starting transition from pediatrics to adult outpatient	− 5.98	− 0.28	− 10.56	− 1.41	0.011
NYHA	3.96	0.19	− 0.70	8.63	0.095
Certification of physical disability	− 1.52	− 0.08	− 5.86	2.81	0.487
Age	4.46	0.22	0.06	8.85	0.047
I take care of myself independently while receiving support	4.17	0.14	− 2.44	10.79	0.213
I am living my own life despite my illness	10.47	0.27	2.26	18.68	0.013
Dependent variable: role/social component summary (RCS)					
Sex	− 6.79	− 0.32	− 11.19	− 2.39	0.003
Presence of family living together	− 2.15	− 0.08	− 8.25	3.95	0.485
Experienced surgery of CHD within last 3 years	− 3.91	3.33	− 10.55	2.73	0.245
Experienced hospitalization within the last 3 years	0.56	0.03	− 4.51	5.63	0.826
Arrythmia	− 0.17	− 0.01	− 5.23	4.89	0.947
Cyanosis	1.71	0.05	− 6.00	9.42	0.660
Starting transition pediatrics to adult	1.05	0.04	− 4.86	6.96	0.726
NYHA	5.97	0.27	1.12	10.87	0.017
Certification of physical disability	− 0.81	− 0.04	− 5.48	3.86	0.732
Age	3.55	0.16	− 1.22	8.33	0.143
I take care of myself independently while receiving support	3.67	0.12	− 3.32	10.67	0.299
I am living my own life despite my illness	9.37	0.22	0.42	18.31	0.040

Univariate linear regression. *N* = 86

*SF-36* the short form 36-item health survey, *B* unstandardized coefficient, *β* standardized coefficient, *CI* confidence interval for *B*, *CHD* Congenital Heart Disease, *NYHA* New York Heart Association

Univariate linear regression analysis (Table 4) identified eight variables significantly associated with PCS, including certification of physical disability (*p* < 0.001) and the presence of cyanosis (*p* = 0.001). For MCS, five variables showed significant associations, including CHD-related surgery within the last 3 years (*p* = 0.009) and initiation of transition from pediatric to adult outpatient care (*p* = 0.011). Three variables were significantly associated with RCS: sex (*p* = 0.003), NYHA classification (*p* = 0.017), and perspectives on “I am living my own life despite my illness” (*p* = 0.040).

### Multiple Linear Regression Analysis on the Scores of the Three SF-36 Components (Table 5)

**Table 5:** Multiple linear regression analysis on the scores of three SF-36 components

Independent variables	*B*	*β*	95% CI		*p*	VIF
			Lower	Upper		
Dependent variable: physical component summary (PCS)						
Arrhythmia	− 5.67	− 0.27	− 9.35	− 1.99	0.003	1.06
Cyanosis	− 7.24	− 0.23	− 12.85	− 1.63	0.012	1.06
Certification of physical disability	− 6.11	− 0.32	− 9.75	− 2.48	0.001	1.78
I take care of myself independently while receiving support	6.39	0.22	1.23	11.56	0.016	1.23
*R*^2^ = 0.391, adjusted *R*^2^ = 0.361, *F* = 13.00, *p* < 0.001						
Dependent variable: mental component summary (MCS)						
Presence of family living together	5.63	0.22	0.52	10.75	0.031	1.02
Experienced CHD surgery within last 3 years	− 9.87	− 0.35	− 15.38	− 4.36	0.001	1.03
I am living my own life despite my illness	11.36	0.29	3.70	19.02	0.004	1.03
*R*^2^ = 0.226, adjusted *R*^2^ = 0.198, *F* = 7.99, *p* < 0.001						
Dependent variable: role/social component summary (RCS)						
Sex	− 6.41	− 0.03	− 10.71	− 2.10	0.004	1.01
NYHA	5.48	0.24	0.84	10.12	0.021	1.01
*R*^2^ = 0.157 adjusted *R*^2^ = 0.136, *F* = 7.71, *p* = 0.001						

Multiple linear regression (stepwise model). *N* = 86

*SF-36* the short form 36-item health survey, *PCS* physical component summary, *MCS* mental component summary, *RCS* role/social component summary, *B* unstandardized coefficient, *β* standardized coefficient, *CI* confidence interval for *B*, *VIF* variance inflation factor, *CHD* congenital heart disease, *NYHA* New York Heart Association

According to the multiple linear regression analysis (Table 5), the presence of arrhythmia and cyanosis, certification of physical disability, and the item “I take care of myself independently while receiving support” explained 36.1% of the variance in PCS (*F* = 13.00, *p* < 0.001). Families living together, the experience of CHD-related surgery within the last 3 years, and “I am living my own life despite my illness,” explained 19.8% of the variance in MCS (*F* = 7.99, *p* < 0.001). Sex and NYHA classification explained 13.6% of the variance in RCS (*F* = 7.71, *p* = 0.001). The effect size, including adjusted *R*^2^ values, revealed modest-to-moderate explanatory power (PCS: 0.36, MCS: 0.20, RCS: 0.14), whereas Cohen’s *f*^2^ effect sizes ranged from small to moderate.

## Discussion

The present study shows that the transition process in Japan often occurs later than recommended, or involves overlapping care between pediatric and adult services, which is commonly regarded as an important and intentional step to support a gradual transition. These observations highlight the need for systematic strategies to ensure timely and coordinated transfer of care for AYAs with CHD. This study, which assessed HRQoL of AYAs with CHD using the SF-36, found overall that physical and mental HRQoL were comparable primarily to the national standard values, whereas role/social HRQoL was lower among females. Multiple regression analyses further revealed clinical severity (arrhythmia, cyanosis, NYHA class, disability certification), cohabitation with family, recent surgical experiences, and subjective transition readiness as associated factors of HRQoL. These findings highlight the interplay between medical, familial, and psychosocial factors in shaping HRQoL when transitioning to adulthood.

Although these models only demonstrated modest explanatory power, as the identified predictors contributed meaningfully to HRQoL. Additional psychosocial and contextual factors not examined in this study are likely to be playing important roles.

### HRQoL in AYAs with CHD: Influence of Sex

Our results showed that the mean PCS and MCS scores of AYAs with CHD were close to the Japanese national standard value of 50 (norm-based scoring, mean = 50, SD = 10, based on the general adult population) [27]. These findings indicated that, at the group level, the physical and mental HRQoL of AYAs with CHD did not differ substantially from the national norms. This result is consistent with prior studies reporting comparable HRQoL scores among AYAs with CHD in other populations [24]. However, females demonstrated notably lower RCS scores, suggesting challenges in social role functioning compared to the general population. The RCS, a concept established through a validation study of the Japanese version, consists of GH, RP, SF, and RE subfactors [34, 35]. In other words, it reflects HRQoL related to social functioning, encompassing physical and mental aspects. Our results showed lower RCS scores for female AYAs with CHD than for males. While we did not directly assess employment status, the finding may be related to those challenges in employment and social participation. In Japan, despite increased female labor force participation from 46.2% in 2012 to 54.4% in 2024, there are still substantial sex disparities [39, 40]. These societal factors may contribute to difficulties in fulfilling expected social roles among female AYAs with CHD. Similarly, in the European Union, the sex gap in employment rates remains [41], indicating that this gap is not unique to Japan.

Besides the generally low employment rate, a survey of 15 high-income countries, including Japan, reported that an overall low employment rate and work limitations for adults with CHD were associated with being female [42]. Although many females with CHD can have successful pregnancies with appropriate specialist care and multidisciplinary support [43, 44], some are advised of considerable risks during pregnancy and childbirth [45], or even regarding sexual activity [46]. In such cases, they may experience heightened anxiety or social isolation. Additionally, females with CHD who face infertility may internalize societal stigma, leading to feelings of inferiority, guilt, and self-blame, which can adversely affect mental health and HRQoL [47]. These findings highlight the importance of comprehensive, interdisciplinary sexuality and reproductive health services tailored to the individual’s medical condition and needs [48].

### Impact of Bodily Pain and Physical Symptoms on HRQoL in AYAs with CHD

Interestingly, bodily pain scores exceed the national standard value in both sexes, especially in males. AYAs with advanced heart disease often experience symptoms like fatigue, shortness of breath, sleep disturbances, and pain during childhood [49]. One possible explanation for these higher scores may be that some AYAs with CHD have become accustomed to living with chronic symptoms and report lower levels of perceived pain. However, this explanation remains speculative, and further research is needed. It is also important to consider that repeated medical procedures and long-term hospitalization may involve medical trauma, which could heighten sensitivity to pain or impact pain perception in complex ways.

A US Congenital Heart Initiative registry study involving over 4500 adults with CHD found that 33% had arrhythmias; despite this, 84% reported a good or better HRQoL [50]. On the contrary, our results indicate that arrhythmia is associated with a low physical HRQoL. Additionally, adults with CHD and cyanosis reported lower HRQoL [51]; our results also suggest an association of cyanosis with lower physical HRQoL. Further, Church et al. [52] suggest that children with physical disabilities have significantly lower HRQoL than non-disabled individuals. In particular, limited functional morbidity and increased pain contribute to lower HRQoL [52]. Similarly, in this study, being AYAs with physical disability due to CHD affected physical HRQoL.

### Supportive Environments for AYAs with CHD in Transitioning to Adulthood

This study addressed two questions related to subjective components of transition readiness to self-reliant adulthood: “I take care of myself independently while receiving support” was positively associated with physical HRQoL. Recently, a randomized controlled trial, the TRANSITION-CHD [53], a transition support program for AYAs with CHD, improved HRQoL; disease knowledge; physical health (cardiopulmonary fitness and physical activity); and mental health (anxiety and depression). Moreover, the STEPSTONES transition program for adolescents with CHD reported that the implementation process was influenced by the adolescents and healthcare professionals engaging with the intervention, as well as the contextual factors [54]. These findings suggest that subjective perceptions of transition readiness may serve as indicators of how well AYAs with CHD are adapting to the challenges of becoming independent adults. This aligns with the goals of structured transition programs, such as TRANSITION-CHD [53] and STEPSTONES [54], which aimed to improve self-management, autonomy, and HRQoL during the transition process. Another study also suggests that social support and higher educational achievement were associated with improved HRQoL [23]. Based on these, not only is it essential during the transition to gain an understanding of one’s illness and knowledge of appropriate treatment behaviors but also that while knowledge is being translated to lifestyle behaviors, environments where one engages and receives support as well as those in which one can do so contribute to the transition and HRQoL. Empowerment through person-centered care develops self-efficacy in patients with chronic conditions; however, this requires shared responsibility between the patient and healthcare professionals [55]. Healthcare professionals’ responsibility entails forming relationships with AYAs with CHD and supporting their transition to adulthood.

### Family Relationships, Autonomy, and Mental HRQoL in AYAs with CHD

Our results also suggest that living with family was associated with better mental HRQoL. Additionally, Im et al. [56] suggest that emotionally warm parental rearing behaviors and siblings were important familial factors positively associated with HRQoL in adolescents with CHD. In contrast, although an emotional bond develops between AYAs and their families, as families grow, the process of independence from them may also become necessary due to the transition into adulthood. Generally, as adolescents age, they become increasingly dependent on and transition support from their mothers, best friends, and romantic partners, and establish independence during adolescence to become emerging adults [57]. This highlights the importance of maintaining moderate dependence and collaborative relationships with family and support groups, rather than striving for complete independence. Self-management is more likely to increase, and HRQoL is better maintained with social support [16]. In this study, participants who endorsed the statement “I am living my own life despite my illness” tended to have higher mental HRQoL. It suggests that fostering a positive self-perception of autonomy, alongside a supportive relationship, may play a crucial role in enhancing mental well-being in AYAs with CHD. In addition, our results revealed the possibility of a lower mental HRQoL in those who underwent CHD-related surgery in the past 3 years. The impact of surgical experiences in children and adolescents with CHD could be explained by the number of surgeries undergone and cardiac condition severity [33]. Long after surgery, it may be necessary to consider both physical and mental issues.

### Employment, Behavioral Limitation, and HRQoL in AYAs with CHD

Finally, our results suggest that, besides the female sex and NYHA factors, accompanying behavioral restrictions may be affected by a lower HRQoL RCS. Disease severity is one factor contributing to unemployment in adult female patients with CHD [58]. Similarly, in adults, Samuel et al. [16, 59] suggest that those in NYHA functional classes III–IV had a lower Work Ability Index than those in NYHA class I. Additionally, adults with complex CHD tend to have lower education and income, as well as lower self-assessments regarding their ability to work. However, flexible work arrangements and workplace accommodation may help maintain these adults in employment. Additionally, females who adopt remote work practice generally report increased satisfaction with their job and life, as well as improved mental health [60]. With increasing research output and as society adopts diverse work styles, adults with CHD may also benefit. Sluman et al. [42] also suggest that female sex and worse NYHA are associated with lower employment opportunities. However, academic background appears to be the primary predictor of successful employment and should be encouraged in children and AYAs with CHD. A transition support program for adolescents with childhood-onset chronic diseases showed effects on transition readiness, self-esteem, and decreased dependence on parents, which may lead to an increased employment rate and social participation [61].

### Limitations

Some participants were unable to complete the questionnaire due to intellectual or physical disability and were excluded. Additionally, a few parents, whose children were unable to self-complete the questionnaire, refused to respond on their children’s behalf. Therefore, the process was not standardized; however, of those with intermediate neurological development impairment, including cognitive impairment, the rate associated with complex presentation was estimated at 20–50% in children with CHD [62]. Thus, there is a need to consider that both included and excluded AYAs are essential to society.

Next, in a prior study, adults with CHD were surveyed to examine the differences in HRQoL by diagnosis [21]. However, in this study, the presence of symptoms, such as arrhythmia and cyanosis, as well as cardiac function classification by NYHA and disability status, was investigated. However, the specific differences resulting from previous diagnoses, treatments, and surgeries were not analyzed.

Finally, this study investigated transition readiness to a self-reliant adulthood using only two questions, based on a literature review and consensus among a small group of researchers. Therefore, there is a need to develop a scale with adequate reliability and validity for determining the readiness status of AYAs with CHD. Additionally, owing to the cross-sectional study design and relatively small sample size from selected sites in Japan, causal inference could not be established, and generalizability is limited. These factors should be considered when interpreting the findings.

## Conclusion

This study demonstrated that the combination of clinical and psychosocial factors influenced HRQoL in AYAs with CHD. Physical HRQoL was linked to clinical severity indicators, including arrhythmia, cyanosis, NYHA functional class, physical disability certification, and subjective readiness (“I take care of myself independently while receiving support”). Mental HRQoL was influenced by recent CHD-related surgery, family cohabitation, and the self-perception of autonomy (“I am living my own life despite my illness”). Moreover, role/social HRQoL was lower in females and in those with behavioral restrictions, reflecting challenges in fulfilling expected social roles. Overall, these findings highlight the importance of comprehensive, sex-sensitive, and family-inclusive approaches to support transition readiness and contribute to better HRQoL in AYAs with CHD.

## Data Availability

The data that support the findings of this study are available from the corresponding author upon reasonable request.

## References

[1] Liu Y, Chen S, Zühlke L, Black GC, Choy MK, Li N, Keavney BD (2019) Global birth prevalence of congenital heart defects 1970–2017: updated systematic review and meta-analysis of 260 studies. Int J Epidemiol 48(2):455–463. 10.1093/ije/dyz009 30783674 10.1093/ije/dyz009 PMC6469300

[2] van der Linde D, Konings EEM, Slager MA, Witsenburg M, Helbing WA, Takkenberg JJM, Roos-Hesselink JW (2011) Birth prevalence of congenital heart disease worldwide: a systematic review and meta-analysis. J Am Coll Cardiol 58(21):2241–2247. 10.1016/j.jacc.2011.08.025 22078432 10.1016/j.jacc.2011.08.025

[3] Stout KK, Daniels CJ, Aboulhosn JA, Bozkurt B, Broberg CS, Colman JM, Crumb SR, Dearani JA, Fuller S, Gurvitz M, Khairy P, Landzberg MJ, Saidi A, Valente AM, Van Hare GF (2019) 2018 AHA/ACC guideline for the management of adults with congenital heart disease: executive summary: a report of the American College of Cardiology/American Heart Association Task Force on Clinical Practice guidelines. J Am Coll Cardiol 73(12):1494–1563. 10.1016/j.jacc.2018.08.1028 30121240 10.1016/j.jacc.2018.08.1028

[4] Mandalenakis Z, Giang KW, Eriksson P, Liden H, Synnergren M, Wåhlander H, Fedchenko M, Rosengren A, Dellborg M (2020) Survival in children with congenital heart disease: have we reached a peak at 97%? J Am Heart Assoc 9(22):e017704. 10.1161/JAHA.120.017704 33153356 10.1161/JAHA.120.017704 PMC7763707

[5] Moons P, Bovijn L, Budts W, Belmans A, Gewillig M (2010) Temporal trends in survival to adulthood among patients born with congenital heart disease from 1970 to 1992 in Belgium. Circulation 122(22):2264–2272. 10.1161/CIRCULATIONAHA.110.946343 21098444 10.1161/CIRCULATIONAHA.110.946343

[6] Downing KF, Nembhard WN, Rose CE, Andrews JG, Goudie A, Klewer SE, Oster ME, Farr SL (2023) Survival from birth until young adulthood among individuals with congenital heart defects: CH strong. Circulation 148(7):575–588. 10.1161/CIRCULATIONAHA.123.064400 37401461 10.1161/CIRCULATIONAHA.123.064400 PMC10544792

[7] Committee for Scientific Affairs, The Japanese Association for Thoracic Surgery, Yoshimura N, Sato Y, Takeuchi H, Abe T, Endo S, Hirata Y, Ishida M, Iwata H, Kamei T, Kawaharada N, Kawamoto S, Kohno K, Kumamaru H, Minatoya K, Motomura N, Nakahara R, Okada M, Saji H, Saito A, Tsuchida M, Suzuki K, Takemura H, Taketani T, Toh Y, Tatsuishi W, Yamamoto H, Yasuda T, Watanabe M, Matsumiya G, Sawa Y, Shimizu H, Chida M (2024) Thoracic and cardiovascular surgeries in Japan during 2021: annual report by the Japanese Association for Thoracic Surgery. Gen Thorac Cardiovasc Surg 72(4):254–291. 10.1007/s11748-023-01997-6 38421591 10.1007/s11748-023-01997-6 PMC10955033

[8] Shiina Y, Toyoda T, Kawasoe Y, Tateno S, Shirai T, Wakisaka Y, Matsuo K, Mizuno Y, Terai M, Hamada H, Niwa K (2011) Prevalence of adult patients with congenital heart disease in Japan. Int J Cardiol 146(1):13–16. 10.1016/j.ijcard.2009.05.032 19493578 10.1016/j.ijcard.2009.05.032

[9] Moons P, Skogby S, Bratt EL, Zühlke L, Marelli A, Goossens E (2021) Discontinuity of cardiac follow-up in young people with congenital heart disease transitioning to adulthood: a systematic review and meta-analysis. J Am Heart Assoc 10(6):e019552. 10.1161/JAHA.120.019552 33660532 10.1161/JAHA.120.019552 PMC8174191

[10] Johnson BH (2016) Promoting patient- and family-centered care through personal stories. Acad Med 91(3):297–300. 10.1097/ACM.0000000000001086 26796094 10.1097/ACM.0000000000001086

[11] Sprong MCA, Zwagerman IR, Soeters L, Slieker MG, Takken T, van den Hoogen A, van Brussel M (2024) Prioritizing family-centered developmental care: insights from parents of children with critical congenital heart disease: a qualitative study. Eur J Pediatr 183(9):3863–3876. 10.1007/s00431-024-05600-9 38888645 10.1007/s00431-024-05600-9 PMC11322194

[12] Uzark K, Wray J (2018) Young people with congenital heart disease: transition to adult care. Prog Pediatr Cardiol 48:68–74. 10.1016/j.ppedcard.2018.01.010

[13] Rosen DS, Blum RW, Britto M, Sawyer SM, Siegel DM, Society for Adolescent Medicine (2003) Transition to adult health care for adolescents and young adults with chronic conditions: position paper of the Society for Adolescent Medicine. J Adolesc Health 33(4):309–311. 10.1016/s1054-139x(03)00208-8 14519573 10.1016/s1054-139x(03)00208-8

[14] Chu PY, Maslow GR, von Isenburg M, Chung RJ (2015) Systematic review of the impact of transition interventions for adolescents with chronic illness on transfer from pediatric to adult healthcare. J Pediatr Nurs 30(5):e19–e27. 10.1016/j.pedn.2015.05.022 26209872 10.1016/j.pedn.2015.05.022 PMC4567416

[15] Gabriel P, McManus M, Rogers K, White P (2017) Outcome evidence for structured pediatric to adult health care transition interventions: a systematic review. J Pediatr 188:263–269.e15. 10.1016/j.jpeds.2017.05.066 28668449 10.1016/j.jpeds.2017.05.066

[16] Sable C, Foster E, Uzark K, Bjornsen K, Canobbio MM, Connolly HM, Graham TP, Gurvitz MZ, Kovacs A, Meadows AK, Reid GJ, Reiss JG, Rosenbaum KN, Sagerman PJ, Saidi A, Schonberg R, Shah S, Tong E, Williams RG, American Heart Association Congenital Heart Defects Committee of the Council on Cardiovascular Disease in the Young, Council on Cardiovascular Nursing, Council on Clinical Cardiology, and Council on Peripheral Vascular Disease (2011) Best practices in managing transition to adulthood for adolescents with congenital heart disease: the transition process and medical and psychosocial issues: a scientific statement from the American Heart Association. Circulation 123(13):1454–1485. 10.1161/CIR.0b013e3182107c56 21357825 10.1161/CIR.0b013e3182107c56

[17] Lee BR, Koo HY, Lee S (2024) Effects of transition programmes to adulthood for adolescents and young adults with CHD: a systematic review with meta-analysis. Cardiol Young 34(5):945–958. 10.1017/S104795112400026X 38525659 10.1017/S104795112400026X

[18] Harada M, Motoki H, Kuwahara K (2025) Transitional care for adult patients with congenital heart disease. Intern Med 64(4):483–491. 10.2169/internalmedicine.4264-24 39111880 10.2169/internalmedicine.4264-24 PMC11904460

[19] Wood DL, Sawicki GS, Miller MD, Smotherman C, Lukens-Bull K, Livingood WC, Ferris M, Kraemer DF (2014) The transition readiness assessment questionnaire (TRAQ): its factor structure, reliability, and validity. Acad Pediatr 14(4):415–422. 10.1016/j.acap.2014.03.008 24976354 10.1016/j.acap.2014.03.008

[20] Uzark K, Smith C, Donohue J, Sunkyung Yu, Afton K, Norris M, Cotts T (2015) Assessment of transition readiness in adolescents and young adults with heart disease. J Pediatr 167(6):1233–1238. 10.1016/j.jpeds.2015.07.043 26298627 10.1016/j.jpeds.2015.07.043

[21] Fteropoulli T, Tyagi M, Hirani SP, Kennedy F, Picaut N, Cullen S, Deanfield JE, Newman SP (2023) Psychosocial risk factors for health-related quality of life in adult congenital heart disease. J Cardiovasc Nurs 38(1):70–83. 10.1097/JCN.0000000000000897 36508238 10.1097/JCN.0000000000000897

[22] Parfeniuk S, Petrovic K, Maclsaac PL, Cook KA, Rempel GR (2020) Transition readiness measures for adolescents and young adults with chronic health conditions: a systematic review. J Transl Med 2(1):1–16. 10.1515/jtm-2020-0020

[23] Harder AT, Mann-Feder V, Oterholm I, Refaeli T (2020) Supporting transitions to adulthood for youth leaving care: consensus based principles. Child Youth Serv Rev 116:105260. 10.1016/j.childyouth.2020.105260

[24] Schrøder M, Boisen KA, Reimers J, Teilmann G, Brok J (2016) Quality of life in adolescents and young adults with CHD is not reduced: a systematic review and meta-analysis. Cardiol Young 26(3):415–425. 10.1017/S104795111500181X 26561207 10.1017/S104795111500181X

[25] Teixeira FM, Coelho RM, Proença C, Silva AM, Vieira D, Vaz C, Moura C, Viana V, Areias JC, Areias MEG (2011) Quality of life experienced by adolescents and young adults with congenital heart disease. Pediatr Cardiol 32(8):1132–1138. 10.1007/s00246-011-0039-0 21710181 10.1007/s00246-011-0039-0

[26] Tatebe S, Yasuda S, Konno R, Sakata Y, Sugimura K, Satoh K, Shiroto T, Miyata S, Adachi O, Kimura M, Mizuno Y, Enomoto J, Tateno S, Nakajima H, Oyama K, Saiki Y, Shimokawa H (2023) Clinical and sociodemographic factors associated with health-related quality of life in patients with adult congenital heart disease—a nationwide cross-sectional multicenter study. Circ J 88(1):62–70. 10.1253/circj.CJ-23-0383 37673658 10.1253/circj.CJ-23-0383

[27] Fukuhara S, Yoshimi S (2004) Manual of SF-36v2, Japanese version. Institute for Health Outcomes and Process Evaluation Research (in Japanese), pp 7–145

[28] Helm PC, Sticker EJ, Keuchen R, Koerten MA, Diller GP, Tutarel O, Bauer UMM (2017) Is having a job a protective factor? Employment status and state of medical care as subjectively perceived by adults with CHD in Germany. Cardiol Young 27(6):1110–1117. 10.1017/S1047951116002146 27830637 10.1017/S1047951116002146

[29] Bottomley A, Pe M, Sloan J, Basch E, Bonnetain F, Calvert M, Campbell A, Cleeland C, Cocks K, Collette L, Dueck AC, Devlin N, Flechtner HH, Gotay C, Greimel E, Griebsch I, Groenvold M, Hamel JF, King M, Kluetz PG, Koller M, Malone DC, Martinelli F, Mitchell SA, Moinpour CM, Musoro J, O’Connor D, Oliver K, Piault-Louis E, Piccart M, Pimentel FL, Quinten C, Reijneveld JC, Schürmann C, Smith AW, Soltys KM, Taphoorn MJB, Velikova G, Coens C, Setting International Standards in Analyzing Patient-Reported Outcomes and Quality of Life Endpoints Data (SISAQOL) consortium (2016) Analysing data from patient-reported outcome and quality of life endpoints for cancer clinical trials: a start in setting international standards. Lancet Oncol 17(11):e510–e514. 10.1016/S1470-2045(16)30510-1 27769798 10.1016/S1470-2045(16)30510-1

[30] Sawatzky R, Kwon JY, Barclay R, Chauhan C, Frank L, van den Hout WB, Nielsen LK, Nolte S, Sprangers MAG, Response Shift – in Sync Working Group (2021) Implications of response shift for micro-, meso-, and macro-level healthcare decision-making using results of patient-reported outcome measures. Qual Life Res 30(12):3343–3357. 10.1007/s11136-021-02766-9 33651278 10.1007/s11136-021-02766-9 PMC8602130

[31] Russell SD, Saval MA, Robbins JL, Ellestad MH, Gottlieb SS, Handberg EM, Zhou Y, Chandler B, HF-ACTION Investigators (2009) New York Heart Association functional class predicts exercise parameters in the current era. Am Heart J 158(4 Suppl):S24–S30. 10.1016/j.ahj.2009.07.017 19782785 10.1016/j.ahj.2009.07.017 PMC2762947

[32] Government of Japan (1970) Act for the welfare of persons with disabilities (Act No. 84 of 1970, as Amended). Ministry of Health, Labour and Welfare. https://www.japaneselawtranslation.go.jp. Accessed 25 June 2025

[33] Latal B, Helfricht S, Fischer JE, Bauersfeld U, Landolt MA (2009) Psychological adjustment and quality of life in children and adolescents following open-heart surgery for congenital heart disease: a systematic review. BMC Pediatr 9:6. 10.1186/1471-2431-9-6 19161602 10.1186/1471-2431-9-6 PMC2642822

[34] Fukuhara S, Bito S, Green J, Hsiao A, Kurokawa K (1998) Translation, adaptation, and validation of the SF-36 health survey for use in Japan. J Clin Epidemiol 51(11):1037–1044. 10.1016/s0895-4356(98)00095-x 9817121 10.1016/s0895-4356(98)00095-x

[35] Fukuhara S, Ware JE, Kosinski M, Wada S, Gandek B (1998) Psychometric and clinical tests of validity of the Japanese SF-36 health survey. J Clin Epidemiol 51(11):1045–1053. 10.1016/s0895-4356(98)00096-1 9817122 10.1016/s0895-4356(98)00096-1

[36] Suzukamo Y, Fukuhara S, Green J, Kosinski M, Gandek B, Ware JE (2011) Validation testing of a three-component model of Short Form-36 scores. J Clin Epidemiol 64(3):301–308. 10.1016/j.jclinepi.2010.04.017 20800993 10.1016/j.jclinepi.2010.04.017

[37] Cohen J (1988) Statistical power analysis for the behavioral sciences, 2nd edn. Psychology Press, Abingdon

[38] Faul F, Erdfelder E, Lang AG, Buchner A (2007) G*power 3: a flexible statistical power analysis program for the social, behavioral, and biomedical sciences. Behav Res Methods 39(2):175–191. 10.3758/bf03193146 17695343 10.3758/bf03193146

[39] Iizuka N (2025) Women’s employment rate nearing highest level since end of war. Jpn Spotlight 2025(March/April):70–73. https://www.jef.or.jp/journal/pdf/260th_Economic_Indicators.pdf

[40] (2024) Japan gender data. World Bank Gender Portal. https://genderdata.worldbank.org/en/economies/japan/

[41] Eurostat (2025) Employment-annual statistics. https://ec.europa.eu/eurostat/statistics-explained/index.php?title=Employment_-_annual_statistics#

[42] Sluman MA, Apers S, Sluiter JK, Nieuwenhuijsen K, Moons P, Luyckx K, Kovacs AH, Thomet C, Budts W, Enomoto J, Yang HL, Jackson JL, Khairy P, Cook SC, Subramanyan R, Alday L, Eriksen K, Dellborg M, Berghammer M, Mattsson E, Mackie AS, Menahem S, Caruana M, Gosney K, Soufi A, Fernandes SM, White KS, Callus E, Kutty S, Bouma BJ, Mulder BJM (2019) Education as important predictor for successful employment in adults with congenital heart disease worldwide. Congenit Heart Dis 14(3):362–371. 10.1111/chd.12747 30714326 10.1111/chd.12747 PMC6849520

[43] Harris RC, Fries MH, Boyle A, Adeniji-Adele H, Cherian Z, Klein N, John AS (2014) Multidisciplinary management of pregnancy in complex congenital heart disease: a model for coordination of care. Congenit Heart Dis 9(6):E204–E211. 10.1111/chd.12163 24447432 10.1111/chd.12163

[44] Lindley KJ, Bairey Merz CN, Asgar AW, Bello NA, Chandra S, Davis MB, Gomberg-Maitland M, Gulati M, Hollier LM, Krieger EV, Park K, Silversides C, Wolfe NK, Pepine CJ (2021) Management of women with congenital or inherited cardiovascular disease from pre-conception through pregnancy and postpartum: JACC focus seminar 2/5. J Am Coll Cardiol 77(14):1778–1798. 10.1016/j.jacc.2021.02.026 33832605 10.1016/j.jacc.2021.02.026 PMC8061781

[45] Galindo MK, Klewer SE, Downing KF, Takamatsu CL, Seckeler MD, Oster ME, Collins RT 2nd, Nembhard WN, Bolin EH, Farr SL (2025) Reproductive health counseling and outcomes among women with congenital heart defects: results from the congenital heart survey to recognize outcomes, needs, and well-being, 2016–2019. Womens Health Issues 35(2):65–73. 10.1016/j.whi.2025.01.005 40023699 10.1016/j.whi.2025.01.005 PMC12017272

[46] Levine GN, Steinke EE, Bakaeen FG, Bozkurt B, Cheitlin MD, Conti JB, Foster E, Jaarsma T, Kloner RA, Lange RA, Lindau ST, Maron BJ, Moser DK, Ohman EM, Seftel AD, Stewart WJ, American Heart Association Council on Clinical Cardiology, Council on Cardiovascular Nursing, Council on Cardiovascular Surgery and Anesthesia, Council on Quality of Care and Outcomes Research (2012) Sexual activity and cardiovascular disease: a scientific statement from the American Heart Association. Circulation 125(8):1058–1072. 10.1161/CIR.0b013e3182447787 22267844 10.1161/CIR.0b013e3182447787

[47] Xie Y, Ren Y, Niu C, Zheng Y, Yu P, Li L (2022) The impact of stigma on mental health and quality of life of infertile women: a systematic review. Front Psychol 13:1093459. 10.3389/fpsyg.2022.1093459 36698573 10.3389/fpsyg.2022.1093459 PMC9869765

[48] Stokes N, Stransky OM, West SC, Hoskoppal A, Talabi MB, Kazmerski TM (2023) Sexual and reproductive health care experiences and perceptions of women with congenital heart disease. Pediatr Cardiol 44(3):564–571. 10.1007/s00246-022-02951-8 35732955 10.1007/s00246-022-02951-8

[49] Blume ED, Kirsch R, Cousino MK, Walter JK, Steiner JM, Miller TA, Machado D, Peyton C, Bacha E, Morell E, American Heart Association Pediatric Heart Failure and Transplantation Committee of the Council on Lifelong Congenital Heart Disease and Heart Health in the Young (2023) Palliative care across the life span for children with heart disease: a scientific statement from the American Heart Association. Circ Cardiovasc Qual Outcomes 16(2):e000114. 10.1161/HCQ.0000000000000114 36633003 10.1161/HCQ.0000000000000114 PMC10472747

[50] Leezer S, Mehta R, Agarwal A, Saraf S, Messmer M, Phillippi R, Jackson JL, Roeder M, Marlin A, Peyser ND, Pletcher MJ, Krasuski R, Lewis M, Reardon L, Saidi A, Kanter R, Sandhu S, Young T, Jacobsen R, Ruckdeschel E, Lubert A, Singh S, Zaidi A, Halpern DH, Mathews A, Carton T, John AS (2024) Patient-reported outcomes among adults with congenital heart disease in the Congenital Heart Initiative Registry. JAMA Netw Open 7(10):e2439629. 10.1001/jamanetworkopen.2024.39629 39412804 10.1001/jamanetworkopen.2024.39629 PMC11581669

[51] Moons P, Luyckx K, Thomet C, Budts W, Enomoto J, Sluman MA, Lu CW, Jackson JL, Khairy P, Cook SC, Chidambarathanu S, Alday L, Eriksen K, Dellborg M, Berghammer M, Johansson B, Mackie AS, Menahem S, Caruana M, Veldtman G, Soufi A, Fernandes SM, White K, Callus E, Kutty S, Ombelet F, Apers S, Kovacs AH (2021) Physical functioning, mental health, and quality of life in different congenital heart defects: comparative analysis in 3538 patients from 15 countries. Can J Cardiol 37(2):215–223. 10.1016/j.cjca.2020.03.044 32739453 10.1016/j.cjca.2020.03.044

[52] Church C, Patil S, Butler S, Miller F, Salazar-Torres JJ, Lennon N, Shrader MW, Donohoe M, Kalisperis F, Mackenzie WGS, Nichols LR (2025) Health-related quality of life of individuals with physical disabilities in childhood. Children (Basel) 12(3):365. 10.3390/children12030365 40150647 10.3390/children12030365 PMC11941727

[53] Bredy C, Werner O, Huguet H, Guillaumont S, Auer A, Requirand A, Lavastre K, Abassi H, De La Villeon G, Vincenti M, Gavotto A, Vincent R, Pommier V, Dulac Y, Souletie N, Acar P, Karsenty C, Guitarte A, Berge M, Marguin G, Masseron MP, Pages L, Bourrel G, Engberink AO, Million E, Huby AC, Leobon B, Picot MC, Amedro P (2024) Efficacy of a transition program in adolescents and young adults with congenital heart disease: the TRANSITION-CHD randomized controlled trial. J Adolesc Health 75(2):358–367. 10.1016/j.jadohealth.2024.04.022 38864791 10.1016/j.jadohealth.2024.04.022

[54] Saarijärvi M, Wallin L, Moons P, Gyllensten H, Bratt EL (2022) Implementation fidelity of a transition program for adolescents with congenital heart disease: the STEPSTONES project. BMC Health Serv Res 22(1):153. 10.1186/s12913-022-07549-7 35123454 10.1186/s12913-022-07549-7 PMC8817652

[55] Pulvirenti M, McMillan J, Lawn S (2014) Empowerment, patient centred care and self-management. Health Expect 17(3):303–310. 10.1111/j.1369-7625.2011.00757.x 22212306 10.1111/j.1369-7625.2011.00757.x PMC5060728

[56] Im YM, Yun TJ, Lee S (2018) Health condition and familial factors associated with health-related quality of life in adolescents with congenital heart disease: a cross sectional study. Health Qual Life Outcomes 16(1):9. 10.1186/s12955-018-0841-y 29321028 10.1186/s12955-018-0841-y PMC5763546

[57] Szwedo DE, Hessel ET, Loeb EL, Hafen CA, Allen JP (2017) Adolescent support seeking as a path to adult functional independence. Dev Psychol 53(5):949–961. 10.1037/dev0000277 28358534 10.1037/dev0000277 PMC5469408

[58] Enomoto J, Mizuno Y, Okajima Y, Kawasoe Y, Morishima H, Tateno S (2020) Employment status and contributing factors among adults with congenital heart disease in Japan. Pediatr Int 62(3):390–398. 10.1111/ped.14152 31957140 10.1111/ped.14152

[59] Samuel BP, Marckini DN, Parker JL, Kay WA, Cook SC (2020) Complex determinants of work ability in adults with congenital heart disease and implications for clinical practice. Can J Cardiol 36(7):1098–1103. 10.1016/j.cjca.2019.11.003 32532555 10.1016/j.cjca.2019.11.003

[60] Agnoletto M (2024) Flexible working and well-being: evidence from the UK. J Demogr Econ 90(4):589–625. 10.1017/dem.2024.16

[61] Morisaki-Nakamura M, Suzuki S, Kobayashi A, Kita S, Sato I, Iwasaki M, Hirata Y, Sato A, Oka A, Kamibeppu K (2022) Efficacy of a transitional support program among adolescent patients with childhood-onset chronic diseases: a randomized controlled trial. Front Pediatr 10:829602. 10.3389/fped.2022.829602 35433550 10.3389/fped.2022.829602 PMC9010051

[62] Marino BS, Lipkin PH, Newburger JW, Peacock G, Gerdes M, Gaynor JW, Mussatto KA, Uzark K, Goldberg CS, Johnson WH Jr, Li J, Smith SE, Bellinger DC, Mahle WT, American Heart Association Congenital Heart Defects Committee, Council on Cardiovascular Disease in the Young, Council on Cardiovascular Nursing, and Stroke Council (2012) Neurodevelopmental outcomes in children with congenital heart disease: evaluation and management: a scientific statement from the American Heart Association. Circulation 126(9):1143–1172. 10.1161/CIR.0b013e318265ee8a 22851541 10.1161/CIR.0b013e318265ee8a

